# Disease Mechanisms and Therapeutic Advances in Idiopathic and Progressive Pulmonary Fibrosis: From Approved Drugs to Emerging Strategies

**DOI:** 10.3390/jcm15114172

**Published:** 2026-05-28

**Authors:** Claudio Tirelli, Giuseppe Muscato, Chiara Alaimo, Irene Di Leo, Sara Mirijaj, Francesco Pennisi, Carlo Vancheri, Michele Mondoni

**Affiliations:** 1 Respiratory Unit, ASST Santi Paolo e Carlo, Department of Health Sciences, Università degli Studi di Milano, 20142 Milan, Italy; 2 Department of Medicine and Surgery, University of Enna “Kore”, 94100 Enna, Italy; 3 Regional Referral Centre for Rare and Interstitial Lung Diseases, University Hospital Policlinico “G. Rodolico-San Marco”, Department of Clinical and Experimental Medicine, University of Catania, 95123 Catania, Italy

**Keywords:** idiopathic pulmonary fibrosis, progressive pulmonary fibrosis, fibrotic niche, epithelial–mesenchymal crosstalk, nerandomilast, admilparant, treprostinil, targeted therapy, antifibrotics

## Abstract

Interstitial lung diseases (ILDs) are frequently characterized by the presence of pulmonary fibrosis (PF), which may lead to respiratory failure secondary to irreversible parenchymal distortion. Although idiopathic pulmonary fibrosis (IPF) is the clinical prototype, the emergence of the progressive pulmonary fibrosis (PPF) phenotype has shifted the therapeutic paradigm toward shared pathogenic pathways while preserving the need to recognize the biological and clinical heterogeneity of the underlying ILDs. This state-of-the-art review integrates recent experimental and clinical data to provide a comprehensive overview of the transition from inflammatory models to the current epithelial-centric framework of fibrogenesis. In particular, the shift from ineffective anti-inflammatory strategies to the success of nintedanib and pirfenidone in both IPF and non-IPF progressive diseases is discussed. Furthermore, recent advances from phase II and III clinical trials targeting specific molecular drivers of fibrosis and vascular remodeling are analyzed, with a focus on pathway-oriented therapies, including nerandomilast, admilparant, and inhaled treprostinil. Understanding this molecular crosstalk is essential for the development of new therapeutic strategy based on precision medicine and it may support a new era of combination approaches aimed at stabilizing disease and improving patient outcomes in both idiopathic and progressive pulmonary fibrosis.

## 1. Introduction

Pulmonary fibrosis (PF) encompasses a heterogeneous group of chronic interstitial lung diseases (ILDs) characterized by progressive scarring of the lung parenchyma, irreversible architectural distortion, and gradual loss of respiratory function [1,2,3]. Despite marked differences in etiology, many fibrotic ILDs share common pathogenic mechanisms, including persistent epithelial injury, dysregulated tissue repair, and excessive extracellular matrix (ECM) deposition [1,4,5,6,7]. These processes ultimately impair gas exchange, worsen dyspnea, and increase mortality [3,8,9]. Idiopathic pulmonary fibrosis (IPF) is the prototypical and most studied fibrotic ILD [8,9]. It is a chronic, progressive fibrosing interstitial pneumonia of unknown cause, usually affecting older adults, and is defined by a radiological and/or histopathological pattern of usual interstitial pneumonia (UIP) [9,10]. However, IPF is not the only fibrotic ILD that may follow a progressive course. Patients with connective tissue disease-associated ILD (CTD-ILD), fibrotic hypersensitivity pneumonitis (HP), sarcoidosis, and unclassifiable interstitial pneumonias may also develop worsening symptoms, progressive physiological impairment, and increasing fibrotic abnormalities on imaging despite appropriate management [1,2,3]. As formalized in the 2022 American Thoracic Society/European Respiratory Society/Japanese Respiratory Society/Asociación Latinoamericana de Tórax (ATS/ERS/JRS/ALAT) clinical practice guideline, this clinical behavior is now defined as progressive pulmonary fibrosis (PPF) [10]. PPF can be diagnosed in non-IPF ILDs with radiological fibrosis and at least two of the following criteria within the previous year, without alternative explanation: worsening respiratory symptoms, physiological and/or radiological progression [3,10]. Depending on the underlying disease, approximately 15–40% of patients with non-IPF fibrotic ILDs develop this phenotype, which is associated with substantial morbidity and mortality [2,3,4]. Recognition of shared pathogenic pathways across fibrotic ILDs has reshaped treatment strategies and supported extension of antifibrotic therapy beyond IPF [4,5,6,10]. In this review, we discuss the pathophysiological basis of fibrogenesis and summarize established pharmacological treatments and emerging therapies currently under phase II and III clinical investigation.

## 2. Materials and Methods

A non-systematic, narrative literature review was conducted. Two search engines (PubMed and Scopus) were used to collect relevant articles from international literature (last access to search engines: 5 March 2026). The search strategy included combinations of the following keywords and was limited to English-language articles: idiopathic pulmonary fibrosis; progressive pulmonary fibrosis; antifibrotic therapy; pirfenidone; nintedanib; nerandomilast; anlotinib; bexotegrast; sufenidone; deupirfenidone; treprostinil; admilparant; clinical trials; epithelial–mesenchymal transition; spirometry; lung function; progression.

Additional eligible studies were retrieved from bibliographies of the most cited studies and book chapters. Data on eligible phase 2 and 3 clinical trials on the topic were obtained from ClinicalTrials.gov. Two authors independently screened abstracts from all the collected articles (C.T.; G.M.); in the case of disagreement, a third senior author screened and decided whether to include it for the analysis. Full texts of the included articles were examined and relevant data summarized.

## 3. Pathophysiology of Pulmonary Fibrosis

**Figure 1:**
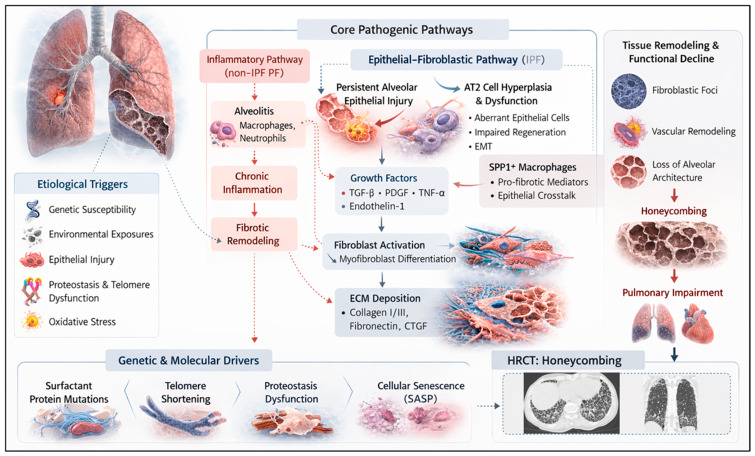
Multicellular mechanisms involved in pulmonary fibrogenesis. Recurrent alveolar epithelial injury, maladaptive epithelial repair, fibroblast recruitment and activation, immune-cell remodeling, extracellular matrix accumulation, vascular dysfunction, cellular senescence, and genetic susceptibility converge to sustain fibrotic progression. IPF, idiopathic pulmonary fibrosis; PF, pulmonary fibrosis; AT2, alveolar type 2; EMT, epithelial–mesenchymal transition; SPP1, secreted phosphoprotein 1; TGF-β, transforming growth factor-β; PDGF, platelet-derived growth factor; TNF-α, tumor necrosis factor-α; ECM, extracellular matrix; CTGF, connective tissue growth factor; SASP, senescence-associated secretory phenotype; HRCT, high-resolution computed tomography.

Despite extensive research efforts, the pathophysiology of PF remains only partially understood. The marked histological, phenotypic, and clinical heterogeneity observed across fibrotic ILDs reflects the complexity of the biological processes that drive the aberrant reparative response of the lung to injury (Figure 1) [1,5,6,7]. Pulmonary fibrosis is currently understood as a multicellular and dynamic process rather than the result of a single dominant pathway. In IPF, the prevailing model emphasizes recurrent alveolar epithelial injury, maladaptive epithelial repair, aberrant alveolar epithelial cell states, fibroblast and myofibroblast activation, immune-cell remodeling, vascular dysfunction, and extracellular matrix accumulation. EMT-related transcriptional programs and partial epithelial plasticity may contribute to this process, but they should be interpreted within the broader context of epithelial–mesenchymal crosstalk rather than as the predominant or sole mechanism. This framework better reflects the complexity of the fibrotic niche, in which epithelial, mesenchymal, immune, vascular, and matrix-derived signals interact to perpetuate aberrant tissue remodeling [5,6,11].

### 3.1. Disease Heterogeneity Within the PPF Framework

PPF should be regarded as a progressive clinical phenotype rather than as a homogeneous disease entity. Indeed, the biological and clinical background of progression may differ substantially across underlying ILDs. In CTD-ILD, fibrosis often develops within a systemic immune-mediated disorder and may be influenced by extrapulmonary disease activity and background immunomodulatory therapy. In fibrotic HP, persistent or recurrent antigen exposure, airway-centered inflammation, and fibrotic remodeling may coexist, making antigen identification and avoidance central components of management. Fibrotic sarcoidosis may show distinct bronchovascular or upper-lobe-predominant involvement and may be complicated by airway distortion and pulmonary hypertension. These differences are clinically relevant because they may influence the balance between inflammation and fibrosis, the role of immunosuppression, timing of antifibrotic therapy and the interpretation of treatment effects across diagnostic subgroups.

### 3.2. Epithelial Injury, Immune Remodeling, and the Fibrotic Niche

In IPF, the weight of evidence no longer supports inflammation as the primary pathogenic trigger; instead, a predominantly epithelial-centric model has emerged [5,9,11]. This view is consistent with the classic histopathological hallmark of temporal heterogeneity, in which preserved lung areas coexist with regions of active fibrosis, inflammatory infiltrates, and honeycomb change [8,9]. Recent experimental and translational studies have strengthened this model by showing that aberrant intermediate alveolar epithelial cells are linked to epithelial detachment, incomplete alveolar regeneration, and pathogenic activation of fibroblasts. These findings indicate that the injured epithelium acts as an active participant in fibrogenesis rather than as a passive victim of tissue damage [11,12].

At the same time, innate immune cells contribute to the maintenance of the fibrotic niche. In particular, secreted phosphoprotein 1-positive (SPP1+) macrophage populations are enriched in fibrotic lung tissue and display a distinctly profibrotic phenotype, promoting fibroblast activation, matrix deposition, and epithelial dysfunction [13,14,15,16,17]. Additional work suggests that osteopontin-related signaling may interact with epithelial stress programs and alternative macrophage activation, thereby amplifying fibrotic remodeling [18,19,20]. Altogether, these findings support the concept that pulmonary fibrosis arises from a dynamic network of epithelial, mesenchymal, and immune-cell interactions [11,21,22,23,24].

### 3.3. Fibroblast Activation and Profibrotic Signaling

Persistent alveolar epithelial injury is therefore considered the key initiating event in pulmonary fibrogenesis. Once activated, alveolar epithelial cells release chemokines and growth factors that stimulate fibroblast recruitment, proliferation, and migration [6,11,21]. In areas of epithelial denudation and basement membrane disruption, fibroblasts accumulate in the subepithelial space, where they continue to proliferate and differentiate into myofibroblasts [21,22,23,24,25]. Myofibroblasts are contractile, highly secretory cells responsible for excessive deposition of collagen and other ECM components, representing an aberrant attempt at tissue repair [23,24,25]. Embedded in a matrix rich in fibrillar type I and III collagens, fibronectin, and other structural proteins, these cells form fibroblastic foci, which are widely regarded as the anatomical and functional sites of active fibrogenesis [24,25,26,27]. Activated fibroblasts release mediators such as angiotensin II, which promote mesenchymal persistence while contributing to apoptosis of adjacent epithelial cells [28].

Progressive loss of alveolar type I cells and capillary endothelium further undermines the integrity of the alveolar–capillary unit [8,9]. This is followed by marked hyperplasia of alveolar type II (AT2) cells, the main progenitor compartment of the distal epithelium; however, under conditions of chronic injury, the regenerative program becomes maladaptive [9,29,30]. AT2 cells adopt transitional and aberrant phenotypes, fail to fully differentiate into alveolar type I cells, and secrete mediators that perpetuate fibrogenesis [11,12,29,30]. These cells produce matrix metalloproteinases involved in ECM remodeling and release profibrotic mediators, including transforming growth factor-β (TGF-β), platelet-derived growth factor (PDGF), tumor necrosis factor- α (TNF-α), and endothelin-1, all of which promote fibroblast proliferation and myofibroblast differentiation [31,32,33,34,35]. Among these pathways, TGF-β is a central regulator of fibrogenesis. It drives myofibroblast differentiation, promotes epithelial dysfunction, and orchestrates transcriptional programs that favor persistent matrix accumulation [32,35,36,37]. Reduced production of endogenous antifibrotic mediators such as prostaglandin E2 (PGE2) removes an important physiological brake on collagen synthesis and fibroblast activation [38,39]. TGF-β signaling also induces connective tissue growth factor (CTGF), which amplifies mesenchymal activation and contributes to persistence of the fibrotic phenotype [40,41,42]. In turn, CTGF promotes broader cytokine and growth factor networks, including vascular pathways involving vascular endothelial growth factor (VEGF), thereby linking matrix remodeling to abnormal vascular responses [40,42,43]. These processes may contribute to vascular remodeling and secondary pulmonary hypertension, particularly when endothelial homeostasis is disrupted [43,44].

Crosstalk among TGF-β, PDGF, complement activation, and additional receptor tyrosine kinase pathways further highlights the complexity and redundancy of profibrotic signaling [33,45,46]. More recently, the lysophosphatidic acid (LPA) pathway has gained attention for its role in lung fibrosis [47,48,49]. Elevated LPA levels, generated by autotaxin, are associated with disease progression and promote fibroblast recruitment, vascular permeability, and profibrotic responses through the LPA1 receptor [47,48,49]. LPA is therefore considered a relevant mediator and potential therapeutic target in pulmonary fibrosis [47,48,49].

### 3.4. Genetic Susceptibility, Epithelial Vulnerability, and Cellular Senescence

Genetic studies have provided further insight into susceptibility to pulmonary fibrosis and into mechanisms that shape disease progression [50]. Genome-wide and sequencing studies have identified variants in genes involved in host defense, epithelial integrity, cell adhesion, mucociliary biology, surfactant homeostasis, and telomere maintenance [50,51,52,53,54]. Importantly, some of these genetic determinants are shared between IPF and non-idiopathic fibrotic ILDs, supporting the concept of partially convergent biological pathways across distinct clinical entities [51,52,54].

Common susceptibility variants are linked to accelerated cellular senescence, aberrant stress responses, and defective proteostasis in alveolar epithelial cells [50,51,52,53,55]. Pathogenic variants in surfactant-related genes, such as SFTPC and SFTPA2, and telomerase-related genes, such as TERT and TERC, are potential monogenic causes of pulmonary fibrosis [50,51,52]. Misfolded surfactant proteins accumulate within the endoplasmic reticulum of AT2 cells, trigger the unfolded protein response, and, once proteostatic capacity is exceeded, induce apoptosis and epithelial attrition [50,55,56]. The resulting AT2 cell dysfunction compromises self-renewal and alveolar regeneration, thereby favoring aberrant repair [11,12,55].

Telomere-related disease represents another major pathogenic axis. Mutations affecting telomerase function lead to premature telomere shortening, epithelial replicative senescence, and acquisition of a senescence-associated secretory phenotype (SASP) enriched in cytokines, chemokines, proteases, and growth factors [50,56,57]. Telomere dysfunction and epithelial senescence have also been linked to genomic instability, oxidative stress, and activation of epithelial–mesenchymal transition programs, further reinforcing the profibrotic microenvironment [56,57,58]. Together, disturbances in proteostasis and telomere biology converge to create epithelial vulnerability, loss of regenerative capacity, and chronic mesenchymal activation [50,55,59].

## 4. Pre-Antifibrotic Era: Historical Perspectives

Historically, treatment of fibrotic ILDs was guided by the inflammatory hypothesis, according to which these disorders were presumed to be predominantly inflammatory, particularly in their early phases, and therefore likely to respond to corticosteroids and immunosuppressive agents [4,8,9]. In the absence of controlled trials, patients with IPF were commonly treated with corticosteroids, cyclophosphamide, azathioprine, or combinations thereof, largely on the basis of anecdotal experience rather than robust evidence [4,8]. This approach was ultimately overturned by randomized data showing harm with combination immunosuppression and no meaningful benefit from antioxidant therapy with N-acetylcysteine, findings that fundamentally reshaped clinical practice [8,59]. The failure of anti-inflammatory strategies redirected attention toward the non-inflammatory drivers of fibrogenesis, including repetitive epithelial injury, aberrant wound healing, and fibroblast activation [5,6,59]. This conceptual shift paved the way for targeted pharmacological approaches and ultimately led to the development of pirfenidone and nintedanib, the first agents shown to slow disease progression in IPF [4,60,61,62,63].

## 5. Pharmacological Treatment for Idiopathic Pulmonary Fibrosis

### 5.1. Pirfenidone

**Figure 2:**
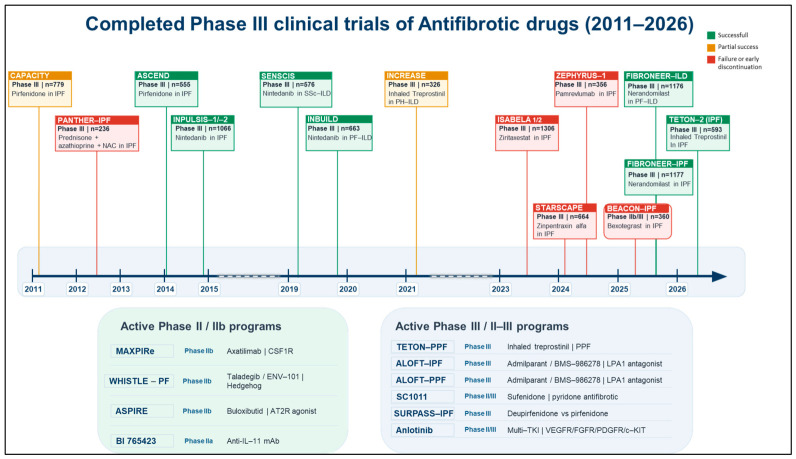
Completed phase III trials and active therapeutic programs in pulmonary fibrosis. Timeline of major completed phase III clinical trials and ongoing phase II (green)–III (blue) programs in idiopathic pulmonary fibrosis (IPF) and progressive pulmonary fibrosis (PPF). Trials included encompass emerging therapies, negative or discontinued programs, and active studies of pathway-oriented agents targeting inflammatory, profibrotic, vascular, extracellular matrix, and receptor tyrosine kinase pathways. (Box representing BEACON-IPF trial differs from others since it was a phase IIb/III trial). IPF, idiopathic pulmonary fibrosis; ILD, interstitial lung disease; PF-ILD, progressive fibrosing interstitial lung disease; PPF, progressive pulmonary fibrosis; SSc-ILD, systemic sclerosis-associated interstitial lung disease; PH-ILD, pulmonary hypertension associated with interstitial lung disease; NAC, N-acetylcysteine; mAb, monoclonal antibody; CSF1R, colony-stimulating factor 1 receptor; LPA1, lysophosphatidic acid receptor 1; IL-11, interleukin-11; VEGFR, vascular endothelial growth factor receptor; FGFR, fibroblast growth factor receptor; PDGFR, platelet-derived growth factor receptor; c-KIT, KIT proto-oncogene receptor tyrosine kinase; TKI, tyrosine kinase inhibitor.

Pirfenidone was the first antifibrotic agent approved for IPF by major regulatory agencies (Table 1, Figure 2). Although its precise mechanism of action remains incompletely defined, it exerts pleiotropic antifibrotic effects, including modulation of TGF-β-driven signaling and inhibition of fibroblast proliferation [4,60,61]. Its clinical efficacy was established in the CAPACITY and ASCEND programs. In the CAPACITY studies, the primary endpoint was change in percent predicted Forced Vital Capacity (FVC) at week 72. Study 004 met this endpoint, showing a significantly smaller decline in percent predicted FVC in the pirfenidone arm than in the placebo group (−8.0% vs. −12.4%; *p* = 0.001), whereas study 006 did not demonstrate a statistically significant difference at week 72 despite an earlier signal favoring active treatment [60,61]. The ASCEND trial subsequently provided confirmatory evidence in a more rigorously selected IPF population. At 52 weeks, pirfenidone reduced the proportion of patients experiencing an absolute decline of at least 10% in predicted FVC or death by 47.9% compared with placebo; it also increased the proportion of patients with no meaningful decline in FVC, reduced the risk of a decline of at least 50 m in the 6 min walk distance, and improved progression-free survival [61]. Pooled analyses of ASCEND and CAPACITY further supported a favorable effect on mortality-related outcomes [60,61]. Pirfenidone is generally well tolerated; the adverse-event profile is dominated by gastrointestinal and cutaneous toxicity, particularly nausea, dyspepsia, anorexia, and photosensitivity, which are generally manageable with slower dose escalation, administration with meals, temporary dose reduction, and rigorous photoprotection [61,63]. Although FVC remains the primary regulatory benchmark, it is increasingly recognized that functional stabilization does not always translate into immediate symptomatic relief. Accordingly, while the ASCEND trial confirmed the effect on FVC, it also highlighted the importance of secondary endpoints such as progression-free survival and reduction in 6MWD decline.

### 5.2. Nintedanib

Nintedanib emerged from the recognition that key pathways involved in fibrogenesis overlap with those driving proliferation and migration in oncology (Table 1, Figure 2). It is an intracellular tyrosine kinase inhibitor targeting receptors for PDGF, VEGF and fibroblast growth factor (FGF), all of which participate in fibroblast activation, migration, and myofibroblast differentiation [4,33,62]. Its efficacy in IPF was demonstrated in the replicate INPULSIS-1 and INPULSIS-2 trials, which together enrolled 1066 patients and consistently met the primary endpoint of reducing the annual rate of FVC decline over 52 weeks [62]. Across the two trials, the between-group difference in FVC decline were 94 and 125 mL/year, respectively, corresponding overall to an approximately 50% reduction in lung function loss versus placebo [62]. Secondary outcomes were more heterogeneous. A lower risk of first acute exacerbation was observed in INPULSIS-2, whereas the same signal was not reproduced with similar strength in INPULSIS-1, prompting a more cautious interpretation of this effect [62]. Nonetheless, the consistency of the primary endpoint across both studies firmly established nintedanib as the second cornerstone antifibrotic therapy in IPF. Clinically, its safety profile is largely defined by gastrointestinal toxicity, particularly diarrhea, which is frequent but usually manageable with antidiarrheal therapy, dose reduction, or brief treatment interruption. Other adverse events include transaminase elevations, which require routine biochemical monitoring [62,63], as well as increased risk of bleeding, that requires particular attention in patients receiving concomitant therapies.

## 6. Antifibrotic Drugs Beyond Idiopathic Pulmonary Fibrosis

The spectrum of pulmonary fibrosis encompasses several conditions beyond IPF, many of which may unfortunately exhibit a similar disease behavior and, consequently, an equally poor prognosis [1,2,3,4]. Although corticosteroids and immunomodulatory agents have long represented the standard therapeutic approach for these disorders, the results obtained with nintedanib and pirfenidone in IPF prompted the scientific community to investigate whether these agents could retain a similar efficacy profile in fibrosing ILDs with a progressive phenotype (Table 1, Figure 2) [4,10]. The first pivotal evidence for antifibrotic therapy in non-IPF fibrotic ILDs came from the SENSCIS trial, which evaluated nintedanib in patients with systemic sclerosis-associated interstitial lung disease (SSc-ILD) [64]. This randomized, double-blind, placebo-controlled trial enrolled 576 patients with SSc-ILD across 32 countries and randomizing them 1:1 to receive nintedanib 150 mg twice daily or placebo for 52 weeks [64]. Notably, approximately half of the patients (48.4%) were receiving mycophenolate at baseline, reflecting real-world clinical practice [64,65]. The results demonstrated that nintedanib significantly reduced the annual rate of FVC decline compared with placebo (−52.4 mL/year vs. −93.3 mL/year; difference 41.0 mL/year; 95% CI, 2.9 to 79.0; *p* = 0.04), representing a 44% relative reduction in the rate of lung function decline [64]. Further analyses showed that nintedanib reduced the risk of absolute decline in FVC of at least 10% predicted or death by 36% (hazard ratio 0.64; 95% CI, 0.43 to 0.95; *p* = 0.029) [65]. Importantly, the treatment effect was consistent regardless of baseline mycophenolate use, with no heterogeneity detected between subgroups, suggesting that the combination of mycophenolate and nintedanib may represent a safe and potentially synergistic treatment option for patients with SSc-ILD [65].

The adverse-event profile was similar to that observed in IPF trials, with diarrhea being the most common adverse event, although more than 80% of patients continued nintedanib for the full 52 weeks [64]. Long-term data from the SENSCIS-ON open-label extension study confirmed that the safety profile remained consistent and that the beneficial effect on FVC decline was maintained over extended treatment periods [66]. The INBUILD study subsequently enrolled 663 patients with ILDs other than IPF who nonetheless showed clear evidence of disease progression despite conventional treatment [67]. Patients were randomized in a 1:1 ratio to receive either nintedanib (150 mg twice daily) or placebo for 52 weeks. A major strength of the study was the rigorous definition of progression required for enrollment, which had to be documented within the previous 24 months: a relative decline in FVC of at least 10% of the predicted value; a decline in FVC between 5% and 10% associated with worsening respiratory symptoms or increased fibrosis extent on high-resolution computed tomography; or worsening respiratory symptoms and radiological progression alone [10,67]. The results showed that nintedanib reduced the annual rate of FVC decline by 57% in the overall population (−80.8 mL/year in the nintedanib group vs. −187.8 mL/year in the placebo group; difference 107.0 mL/year; 95% CI, 65.4 to 148.5; *p* < 0.001) [67]. Importantly, the treatment effect was consistent across all subgroups, regardless of the underlying clinical diagnosis or radiological pattern [67]. However, the apparent consistency of treatment effect across diagnostic subgroups should be interpreted with caution. PPF trials are generally designed and powered to detect treatment effects in the overall progressive fibrotic population, whereas individual diagnostic subgroups are often smaller and biologically heterogeneous. Therefore, the absence of statistically significant heterogeneity does not necessarily imply identical mechanisms of progression or identical treatment responsiveness across different conditions (e.g., CTD-ILD, fibrotic HP, unclassifiable ILD). Future studies should therefore consider stratification according to underlying diagnosis, radiological pattern, background immunomodulatory therapy, and predefined progression criteria. These findings provided the scientific basis for extending the regulatory indications of nintedanib. Indeed, the concept of progressive pulmonary fibrosis (PPF) was formalized thereafter by the 2022 ATS/ERS/JRS/ALAT clinical practice guideline, which for the first time suggested the use of an antifibrotic drug, nintedanib, in fibrotic ILDs other than IPF on the basis of the INBUILD findings [10]. Furthermore, real-life studies confirmed the safety and effectiveness of nintedanib in slowing lung function decline, regardless of etiological diagnosis and radiological pattern [68,69,70].

Evidence for pirfenidone beyond IPF remains more limited and less uniform. Several phase II studies have attempted to extend its indication to non-IPF fibrosing ILDs, but the overall dataset remains constrained by small sample size, heterogeneity, and premature trial discontinuation. The RELIEF trial, which evaluated the addition of pirfenidone to conventional therapy in patients with non-IPF progressive fibrotic ILDs, was terminated prematurely for futility due to slow recruitment, which limited its statistical power. Consequently, the study failed to reach its primary endpoint in the intention-to-treat population. Nevertheless, exploratory analyses suggested a potential attenuation of FVC decline in patients receiving pirfenidone (mean difference 89 mL; *p* = 0.042), although these findings must be interpreted with caution because of the trial’s early termination. and limited power to draw definitive conclusions on pirfenidone’s efficacy in non-IPF ILDs [71]. In the phase II TRAIL1 study in rheumatoid arthritis-associated ILD (RA-ILD), the primary composite endpoint of significant FVC decline or death was not met, largely because the study became underpowered after early termination. However, secondary analyses indicated a slower rate of FVC loss with pirfenidone, particularly in patients with more extensive fibrosis or a UIP-like pattern [72].

Real-world data have broadly aligned with these observations, suggesting relative stabilization of lung function after antifibrotic initiation in selected RA-ILD populations [73,74]. Current guidelines recommendations therefore remain more cautious for pirfenidone than for nintedanib in non-IPF disease. Recent ACR/CHEST (American College of Rheumatology/American College of Chest Physicians), ATS, and ERS/EULAR (European Alliance of Associations for Rheumatology) guidance supports nintedanib in progressive systemic autoimmune rheumatic disease-associated ILD, especially in fibrosing phenotypes, while pirfenidone is generally considered a more selective option in RA-ILD progression or in settings where individualized decision-making is warranted [75,76,77].

## 7. From Setbacks to Breakthrough: The Emergence of Nerandomilast in Antifibrotic Therapy

Despite the approval of pirfenidone and nintedanib, progress in antifibrotic drug development remained limited for more than a decade (Figure 2). Several late-stage programs failed to confirm earlier promise, including ziritaxestat (a selective inhibitor of autotaxin) in the ISABELA trials, zinpentraxin alfa (a recombinant form of human pentraxin-2) in STARSCAPE, and pamrevlumab (an inhibitor of the CTGF—co-factor and amplifier of TGF-β) in ZEPHYRUS-1 [78,79,80,81]. These setbacks highlighted both the biological complexity of fibrotic lung disease and the difficulty of demonstrating efficacy beyond slowing FVC decline [4,78]. Against this background, the FIBRONEER program marked a major advance by showing efficacy of nerandomilast in both IPF and PPF [82,83] (Figure 2). Nerandomilast is a selective phosphodiesterase 4B (PDE4B) inhibitor and the first agent in this class to reach clinical practice in IPF (Table 1) [79,81,82,83]. Unlike existing antifibrotics, it enhances intracellular cyclic AMP signaling through selective PDE4B inhibition, thereby modulating both inflammatory and fibrotic pathways [84,85,86,87]. This mechanism reduces release of profibrotic mediators, attenuates fibroblast-to-myofibroblast differentiation, and decreases cell contractility and migration [85,87,88]. Preclinical studies also suggest favorable effects on endothelial barrier dysfunction and on broader signaling networks, including TGF-β—and Mitogen-Activated Protein Kinases (MAPK)-related pathways [87,88]. Isoform selectivity was specifically pursued to improve tolerability compared with non-selective PDE4 inhibitors [84,85]. In the phase III FIBRONEER-IPF trial, nerandomilast significantly reduced FVC decline at 52 weeks compared with placebo [82]. Specifically, adjusted mean changes in FVC at week 52 were −114.7 mL (95% CI, −141.8 to −87.5) in the nerandomilast 18 mg group, −138.6 mL (95% CI, −165.6 to −111.6) in the 9 mg group, and −183.5 mL (95% CI, −210.9 to −156.1) in the placebo group [82]. The adjusted difference versus placebo was 68.8 mL (95% CI, 30.3 to 107.4; *p* < 0.001) for nerandomilast 18 mg and 44.9 mL (95% CI, 6.4 to 83.3; *p* = 0.02) for nerandomilast 9 mg [82].

Importantly, benefit was observed both in patients receiving background pirfenidone or nintedanib and in those treated without background antifibrotic therapy, supporting a role for nerandomilast as either add-on or stand-alone treatment [82]. However, a pharmacokinetic interaction between nerandomilast and pirfenidone was identified, reducing plasma concentrations of nerandomilast by approximately 50% in patients taking pirfenidone; consequently, only the 18 mg twice-daily dose of nerandomilast appeared to be effective in these patients [82]. A more cautious interpretation is required regarding harder clinical endpoints. Analysis of the complete follow-up period in FIBRONEER-IPF showed no significant effect on the composite endpoint of time to first acute exacerbation, hospitalization for respiratory cause, or death, although nerandomilast 18 mg twice daily was associated with a numerically lower risk of death, though not reaching statistical significance (hazard ratio 0.66; 95% CI, 0.41 to 1.08) [85]. Furthermore, although PDE4B inhibition aimed to improve tolerability, gastrointestinal events and weight loss remained common, potentially affecting long-term adherence in a real-world setting.

In parallel with the IPF program, the phase III FIBRONEER-ILD trial evaluated nerandomilast in patients with PPF using a similar study design [83]. This trial also met its primary endpoint, demonstrating that nerandomilast significantly reduced FVC decline compared with placebo over 52 weeks. The adjusted mean change in FVC at week 52 was −98.6 mL (95% CI, −123.7 to −73.4) in the nerandomilast 18 mg group, −84.6 mL (95% CI, −109.6 to −59.7) in the 9 mg group, and −165.8 mL (95% CI, −190.5 to −141.0) in the placebo group [80]. The adjusted difference versus placebo was 67.2 mL (95% CI, 31.9 to 102.5; *p* < 0.001) for nerandomilast 18 mg and 81.1 mL (95% CI, 46.0 to 116.3; *p* < 0.001) for nerandomilast 9 mg [83]. Subgroup analyses suggested a generally consistent effect of nerandomilast across different ILD diagnoses, including CTD-ILD, fibrotic HP, and other fibrosing ILDs; nerandomilast also slowed lung function decline both in patients taking background nintedanib therapy and in those not receiving such therapy [83]. The safety profile of nerandomilast appears manageable. Diarrhea was the most common adverse event across FIBRONEER studies, but it was usually mild to moderate and led only infrequently to permanent discontinuation [82,83,89]. Other adverse events included weight loss, reduced appetite, nausea, and mood-related symptoms, generally at rates comparable to or only slightly higher than placebo [82,89].

From a mechanistic standpoint, the dual anti-inflammatory and antifibrotic activity of PDE4B inhibition may be particularly attractive in progressive fibrotic ILDs, where immune dysregulation and fibrogenesis frequently coexist [85,87,88]. More broadly, the emergence of nerandomilast has re-energized the field and suggests that future drug development may increasingly focus on combination strategies, mechanistically complementary agents, and better phenotypic or molecular enrichment of clinical trials [78,84,85,86,87]. Nevertheless, combination antifibrotic therapy should be interpreted as a promising but still unresolved strategy. Potential drug–drug interactions may influence exposure and dosing, as shown by the interaction between nerandomilast and pirfenidone [82]. In addition, overlapping adverse-event profiles, including gastrointestinal intolerance, weight loss, liver enzyme abnormalities, and treatment discontinuation, may increase cumulative toxicity and affect long-term adherence. Combination regimens also raise trial-design and regulatory challenges, because future studies will need to distinguish monotherapy effects from true additive benefit on top of approved background treatment. Careful stratification by concomitant antifibrotic or immunomodulatory therapy will therefore be essential before these strategies can be translated into routine clinical practice.

## 8. Pathway-Driven Emerging Therapies

In recent years, knowledge of the molecular and cellular basis that drive fibrotic lung remodeling has substantially increased. As a consequence, a new generation of pathway-oriented therapeutic strategies have been investigated, and some molecules are now being tested in phase II and III clinical trials [90]. These drugs are designed to target specific pathogenic axes, ranging from aberrant fibroblast activation and extracellular matrix deposition to dysregulated vascular signaling, inflammation, and epithelial injury responses (Table 2, Figure 2).

### 8.1. Admilparant (BMS-986278)

Enhanced lysophosphatidic acid receptor 1 (LPA1) signaling has been implicated in the fibrotic remodeling and progressive lung function decline in both idiopathic pulmonary fibrosis (IPF) and progressive pulmonary fibrosis (PPF) [47]. Admilparant (BMS-986278) is a selective, orally administered LPA1 antagonist developed to inhibit this profibrotic signaling pathway by reducing fibroblast migration and collagen synthesis, thereby supporting its potential therapeutic application in pulmonary fibrosis [47]. A phase 2, randomized, double-blind, placebo-controlled trial (NCT04308681) enrolled parallel cohorts of patients with IPF (*n* = 278 randomized; *n* = 276 treated) and PPF (*n* = 125 randomized; *n* = 123 treated) [91]. Participants were assigned to receive admilparant 30 mg or 60 mg, or placebo, twice daily for 26 weeks. Concomitant antifibrotic therapy was permitted in both cohorts, whereas immunosuppressive therapy was allowed only in the PPF subgroup.

The findings demonstrated that the 60 mg dose of admilparant reduced the rate of decline in percent predicted forced vital capacity (FVC) compared to placebo in both IPF and PPF cohorts. Specifically, the treatment difference in FVC change versus placebo was 62 mL (95% CI, −1 to 125) for the IPF cohort and 113 mL (95% CI, 27 to 199) for the PPF cohort over 26 weeks. Admilparant also delayed time to disease progression, with hazard ratios of 0.54 (IPF) and 0.41 (PPF) for the 60 mg dose versus placebo, indicating a clinically meaningful effect. Overall, admilparant demonstrated a safety and tolerability profile similar to placebo [91]. Among all study cohorts, diarrhea was the most frequently reported treatment-emergent adverse event (TEAE), occurring predominantly among participants receiving concomitant antifibrotic therapy. Within the IPF cohort, diarrhea incidence rates among patients receiving background antifibrotic medications were 16% in the placebo arm, 13% in the 30 mg treatment group, and 13% in the 60 mg treatment group. Among participants not receiving background antifibrotic therapy, diarrhea rates were 3% for placebo, 7% for 30 mg, and 7% for 60 mg. In the PPF cohort, diarrhea occurred in 40%, 13%, and 13% of patients receiving placebo, 30 mg, and 60 mg respectively among those on background antifibrotic therapy. Among participants without concomitant antifibrotic treatment, diarrhea incidence was 0% for placebo, 16% for 30 mg, and 4% for 60 mg. Transient decreases in blood pressure were observed after the first dose; however, these changes were not clinically significant and were not associated with higher rates of treatment discontinuation or serious adverse events. In the IPF subcohort, the incidence was 1.1%, 2.2%, and 4.3% in the placebo, 30 mg, and 60 mg groups, respectively. In the PPF subcohort, corresponding rates were 0%, 10%, and 7.1% respectively for placebo, 30 mg, and 60 mg [91].

Notably, in a post hoc analysis, the 60 mg dose was associated with a prolonged time to disease progression in IPF (hazard ratio 0.54; 95% CI 0.31–0.95), where progression was defined as a composite outcome including one or more of the following events: ≥10% relative FVC decline, acute exacerbation, hospitalization, or death [92]. This effect was consistent across subgroups, irrespective of baseline lung function or background antifibrotic therapy. By contrast, the 30 mg dose attenuated FVC decline in PPF (−2.9% vs. −4.3% with placebo) but had no effect in IPF (−2.8% vs. −2.7%) [92]. Although admilparant was overall well tolerated, the long-term impact on systemic blood pressure and the efficacy of the lower 30 mg dose in IPF remain areas of uncertainty. Two phase 3 studies are currently underway to evaluate the efficacy, safety, and tolerability of admilparant in IPF (ALOFT-IPF; NCT06003426) and PPF (ALOFT-PPF; NCT06025578). Both trials share co-primary endpoints, including the incidence of spontaneous syncopal events at approximately 4 weeks and the absolute change from baseline in FVC (mL) through week 52.

### 8.2. Inhaled Treprostinil

Treprostinil is a synthetic analog of prostacyclin with well-established pharmacodynamic properties, including direct vasodilation of the pulmonary and systemic arterial circulation and inhibition of platelet aggregation [93]. Although initially approved for the treatment of World Health Organization (WHO) Group 1 pulmonary arterial hypertension (PAH), treprostinil has also proven effective also in WHO Group 3 pulmonary hypertension (PH), particularly among patients with interstitial lung disease (ILD). Consequently, inhaled treprostinil has gained attention as a promising therapeutic option for ILD-associated pulmonary hypertension (PH-ILD), warranting continued investigation. The INCREASE trial was a pivotal, 16-week, multicenter, randomized, double-blind, placebo-controlled study enrolling 326 patients with ILD and hemodynamically confirmed PH by right heart catheterization [94]. Participants were randomized to receive inhaled treprostinil (up to 12 breaths, 72 μg, four times daily) or placebo.

The trial met its primary endpoint, demonstrating a statistically significant improvement in 6 min walk distance (6MWD), with a least-squares mean difference of 31.12 m in favor of treprostinil (95% CI 16.85–45.39; *p* < 0.001). Key secondary endpoints were also successfully achieved, including a 15% reduction from baseline in NT-proBNP levels in the treprostinil arm compared with a 46% increase in the placebo arm (treatment ratio 0.58; 95% CI 0.47–0.72; *p* < 0.001). Additionally, clinical worsening events were significantly reduced (22.7% vs. 33.1%; HR 0.61; 95% CI 0.40–0.92; *p* = 0.04). The safety profile was consistent with prior prostacyclin studies, with cough, headache, dyspnea, dizziness, nausea, fatigue, and diarrhea reported most frequently [94]. The inhaled treprostinil program provided an example of moving beyond FVC. The INCREASE trial utilized 6MWD as its primary endpoint to directly assess exercise capacity, while also demonstrating a significant reduction in clinical worsening events and NT-proBNP levels, offering a more holistic view of patient stability than FVC alone.

A post hoc analysis of the INCREASE trial revealed unexpected improvements in pulmonary function, particularly among patients with idiopathic interstitial pneumonia, and most notably in those with idiopathic pulmonary fibrosis (IPF) [95]. In the IPF subgroup (*n* = 92), inhaled treprostinil produced a placebo-corrected mean improvement in forced vital capacity (FVC) of 168.5 mL at week 16 (95% CI 40.1–297.0; *p* = 0.011), although the difference at week 8 did not reach statistical significance (84.5 mL; 95% CI −20.4 to 189.5; *p* = 0.11). The percentage predicted FVC also improved significantly: 2.5% (95% CI 0.1–4.9; *p* = 0.038) at week 8 and 3.5% (95% CI 0.7–6.3; *p* = 0.015) at week 16. Notably, 53% of patients with IPF were receiving background antifibrotic therapy, suggesting that the observed improvements in FVC may be additive to existing standard-of-care treatments [95].

Further insight into the long-term effects of inhaled treprostinil was provided by a survival analysis combining data from the INCREASE trial and its open-label extension (OLE) [96,97]. In this extension study, 29 deaths were reported among participants originally randomized to treprostinil and 33 among those initially assigned to placebo. Standard, unadjusted analyses demonstrated a non-significant trend toward reduced mortality (HR 0.71; 95% CI 0.46–1.10; *p* = 0.12). However, after adjustment for treatment crossover, a significant reduction in mortality was observed. These findings suggested a possible long-term survival benefit of inhaled treprostinil in patients with PH-ILD [96,97]. Recent evidence from phase 3 clinical trials has suggested that inhaled treprostinil may exert antifibrotic effects independently of its known vasodilatory properties. The international TETON-2 trial (NCT05255991) evaluated whether these effects translate into meaningful clinical benefit in patients with ILD even in the absence of PH [98]. In this randomized, double-blind, placebo-controlled study, inhaled treprostinil met its primary endpoint, demonstrating a statistically significant improvement in the absolute change in FVC from baseline to week 52. The mean difference between the inhaled treprostinil group and the placebo group was 95.6 mL (95% CI: 52.2 to 139.0 mL; *p* < 0.001). Furthermore, the risk of clinical worsening or death was significantly reduced in the active treatment arm (HR: 0.71; 95% CI: 0.53 to 0.95; *p* = 0.02) [98]. These results support the potential role of inhaled treprostinil as a new effective therapeutic option in fibrotic lung disease. Inhaled treprostinil was generally well tolerated, although treatment discontinuations were reported. Overall discontinuation rates were 33.6% in the treprostinil group and 24.7% in the placebo group. The most frequently reported adverse event was cough, occurring in 48.3% of patients receiving treprostinil compared with 24.1% in the placebo group [98]. Thus, despite the promising FVC data, considerations about safety and tolerability remain central. The high incidence of cough (48.3% vs. 24.1% in placebo) and a discontinuation rate of 33.6% highlight the challenges of inhaled administration in patients with already compromised lung function. Recently, topline results from the TETON-1 phase III trial have been announced and confirmed the efficacy of inhaled treprostinil in a distinct cohort of IPF patients. The study met its primary endpoint, showing a statistically significant reduction in the rate of FVC decline compared with placebo, consistent with the findings observed in TETON-2. These results further confirm the potential of inhaled prostacyclin analogs as a novel therapeutic avenue in IPF management. The ongoing TETON-PPF study (NCT05943535) is currently evaluating whether these clinical benefits extend to patients with progressive pulmonary fibrosis (PPF) other than IPF. An accompanying open-label extension, TETON-OLE (NCT04905693), will evaluate the long-term safety and tolerability of inhaled treprostinil in patients with IPF or PPF for up to six years, or until early discontinuation due to adverse events, drug discontinuation for other reasons, commercial availability of inhaled treprostinil for IPF or PPF, or study termination by the sponsor.

### 8.3. Bexotegrast (PLN-74809)

Bexotegrast (PLN-74809) is an antifibrotic agent designed to inhibit fibrogenesis through dual-selective antagonism of the TGF-β-activating integrins αvβ6 and αvβ1. By preventing integrin-mediated activation of latent TGF-β, bexotegrast attenuates downstream profibrotic signaling pathways implicated in extracellular matrix deposition and progressive parenchymal remodeling in idiopathic pulmonary fibrosis (IPF) [99]. The phase 2a INTEGRIS-IPF trial was a randomized, double-blind, placebo-controlled, dose-ranging, multicenter study evaluating the safety, tolerability, and pharmacokinetics of bexotegrast in patients with IPF [100]. Participants were randomized to receive oral bexotegrast once daily at doses of 40, 80, 160, or 320 mg or placebo, administered either as monotherapy or in combination with standard antifibrotic therapy (pirfenidone or nintedanib). Within each cohort, patients were assigned in an approximate 3:1 ratio (bexotegrast:placebo) and treated for a minimum of 12 weeks. The primary endpoint was the incidence of treatment-emergent adverse events (TEAEs). The overall safety profile of bexotegrast was comparable to placebo (69.7% [62/89] vs. 67.7% [21/31], respectively), with most TEAEs classified as mild or moderate in severity. Diarrhea was the most frequently reported adverse event, predominantly among participants receiving concomitant nintedanib, suggesting a potential additive gastrointestinal effect rather than a distinct drug-specific toxicity signal [100]. Beyond safety, exploratory efficacy analyses demonstrated a reduced decline in FVC among patients treated with bexotegrast compared with placebo (mean changes in FVC from baseline to week 12 were 46.1 mL, 25.6 mL, 25.6 mL, and 29.4 mL for the 40, 80, 160, and 320 mg bexotegrast groups, respectively, compared with 110.5 mL in the placebo group), irrespective of background antifibrotic therapy [100]. Quantitative lung fibrosis imaging revealed a dose-dependent attenuation of fibrotic progression on high-resolution computed tomography (HRCT), and reductions in circulating fibrosis-associated biomarkers were observed in the active-treatment groups [100]. Collectively, these findings provide preliminary evidence of biological and functional antifibrotic activity consistent with the proposed mechanism of TGF-β pathway modulation.

In a separate phase 2, double-blind, placebo-controlled imaging study (NCT05621252), 12 weeks of bexotegrast 160 mg once daily reduced active type I collagen deposition in adults with IPF, as assessed by 68Gallium-Collagen Binding Probe 8 positron emission tomography (68Ga-CBP8 PET) [101]. Notably, the imaging studies revealed that antifibrotic effects were most pronounced in subpleural lung regions, which are typically the first to be involved in IPF. Although limited by small sample size (*n* = 10; 7 bexotegrast, 3 placebo), the study suggested favorable effects on lung remodeling and microvascular architecture [101]. Mechanistic target engagement was further evaluated in the phase 2, open-label study (NCT04072315), which assessed αvβ6 integrin receptor occupancy using PET imaging following single oral doses of bexotegrast (60–320 mg) in patients with IPF [102]. Receptor occupancy increased in a dose-dependent manner, reaching up to 92% at the highest dose tested [102]. No treatment-emergent adverse events attributable to bexotegrast were reported, supporting the pharmacodynamic activity of the compound and informing the selection of once-daily doses of 160–320 mg for subsequent efficacy trials.

Subsequently, the phase 2b/3 BEACON-IPF trial (NCT06097260), a randomized, double-blind, placebo-controlled study, was initiated to evaluate the long-term efficacy and safety of bexotegrast in IPF [103]. The primary endpoint was the change from baseline in absolute FVC (mL) at week 52. Collectively, early-phase clinical investigations of bexotegrast demonstrated a favorable safety profile, substantial target engagement, and consistent signals of antifibrotic activity across functional, imaging, and biomarker endpoints [103]. While these data support continued exploration of integrin-mediated TGF-β inhibition as a therapeutic strategy in IPF, the clinical implications of longer-term FVC outcomes were not fully realized, as the BEACON-IPF trial was terminated early due to safety concerns identified by the Data and Safety Monitoring Board (DSMB) [103]. In this sense, bexotegrast trials can be considered a critical reminder of the volatility in the development of antifibrotic drugs, which is often characterized by encouraging phase II findings that arenot confirmed in subsequent phase III trials. This again underscores the need for rigorous, long-term monitoring of investigational therapies.

### 8.4. Sufenidone (SC1011)

Sufenidone (SC1011) is a novel pyridone derivative rationally designed to enhance antifibrotic and anti-inflammatory activity while addressing the pharmacological limitations of first-generation compounds [90]. Preclinical investigations demonstrated that sufenidone exerts more potent inhibitory effects on fibrogenic and inflammatory pathways than the earlier-generation compound pirfenidone, while maintaining favorable pharmacokinetic properties and good tolerability across multiple animal species [104]. These findings supported its transition into clinical development and a potential next-generation antifibrotic agent for IPF. Consequently, a randomized, double-blind, placebo-controlled combined phase 2/3 clinical trial (NCT06125327) was initiated to evaluate the efficacy and safety of sufenidone in patients with IPF. The study used an adaptive design, featuring an interim analysis at 26 weeks to determine the optimal dose, followed by a continuation to a total treatment duration of 52 weeks with an additional 4-week safety follow-up. The primary endpoint was defined as the annual rate of decline in FVC, used as a surrogate marker of disease progression. Recent clinical data from the trial indicate that sufenidone significantly attenuates the FVC decline compared with placebo. Furthermore, the drug demonstrated superior tolerability, notably showing a reduced incidence of gastrointestinal distress and skin photosensitivity, the most common dose-limiting toxicities associated with earlier pyridone therapies.

### 8.5. Deupirfenidone (LYT-100)

Deupirfenidone (LYT-100) is a selectively deuterated formulation of pirfenidone engineered to achieve a differentiated pharmacokinetic profile that enables higher plasma exposure, with the aim of improving both tolerability and antifibrotic potency in the treatment of IPF [105]. The ELEVATE trial was a phase 2b, international, multicenter, randomized, double-blind, active- and placebo-controlled dose-finding study designed to evaluate the efficacy, tolerability, safety, and dose response of deupirfenidone 550 mg TID and 825 mg TID compared with pirfenidone 801 mg TID and placebo in 257 treatment-naive adult patients with IPF from 14 countries [105]. The primary endpoint was the rate of change in forced vital capacity (FVC, mL) over 26 weeks, with the rate of change in percent predicted FVC (FVCpp) as the key secondary endpoint being. Using a Bayesian analysis, the posterior probability that the pooled deupirfenidone arms had less FVC decline than placebo was 98.5% for absolute FVC and 99.6% for FVCpp, thereby meeting both the primary and key secondary endpoints. A clear dose response was demonstrated between the 550 mg and 825 mg doses, with the 825 mg dose also achieving statistical significance by frequentist analysis and producing an FVC decline approaching the level seen with normal aging. Deupirfenidone was well tolerated at both doses, with a safety profile consistent with previous studies; notably, the incidence of gastrointestinal adverse events (nausea, dyspepsia, diarrhea) and photosensitivity reactions was numerically lower in the deupirfenidone arms than in the pirfenidone arm [106]. Building on these results, the SURPASS-IPF study, a randomized, double-blind, head-to-head phase 3 trial, was initiated to extend the primary outcome to 52 weeks of treatment and demonstrate the superiority of deupirfenidone over pirfenidone in participants with IPF who are not receiving background therapy.

### 8.6. Anlotinib

Anlotinib is an oral, multitarget tyrosine kinase inhibitor primarily adopted in oncology, for the treatment of non-small-cell lung cancer and soft tissue sarcoma [90]. In preclinical models, anlotinib demonstrated not only antiangiogenic activity but also significant antifibrotic effects by inhibiting VEGFR, PDGFR, and FGFR signaling, thereby suppressing myofibroblast activation and reducing collagen deposition through the modulation of glycolytic pathways [90]. An ongoing Phase 2 clinical trial (NCT05828953) is currently evaluating the safety and efficacy of anlotinib capsules in patients with IPF and other progressive fibrosing interstitial lung diseases (PF-ILDs). The study focuses on the change in FVC over a 24-week period as its primary endpoint, aiming to determine whether anlotinib can provide a multi-pathway inhibitory approach distinct from existing antifibrotic therapies.

## 9. Future Perspectives: From Genetic Stratification to Senescent Cell Clearance

The treatment of PF is currently approaching new molecular targets and pathophysiological mechanisms, including inflammatory drivers and vascular compartment. Future perspectives may also include genetics and epigenetics, with the aim of developing a more tailored approach based on specific variants. To date, the role of genetic influences in PF remains a matter of debate. Although rare variants in telomerase-related genes (TRGs) or surfactant-related genes (SRGs) are associated with worse prognosis, targeted therapies are lacking and data remain inconclusive regarding the real effects of current antifibrotics have in patients with Familial Pulmonary Fibrosis [51,87]. Based on current knowledge, identification of common variants may in the future serve as a reliable marker of disease risk and therapeutic efficacy. The MUC5B promoter variant (rs35705950) remains the most significant genetic risk factor and a potential biomarker for treatment stratification. Although this variant is associated with a marked increase in mucin production in the distal airways, patients carrying it may exhibit a distinct “mucin-high” phenotype that could respond differently to mucolytic or immunomodulatory strategies [107].

Interestingly, a post hoc analysis of the PANTHER-IPF trial showed substantial benefit from N-acetylcysteine (NAC) therapy in patients with the TT genotype of the TOLLIP (Toll-interacting protein) rs3750920 variant [108,109]. More recently, the concept of polygenic risk scores (PRSs) has emerged [110]. PRS aggregate the effects of variants in several genes to predict the trajectory of progression in pulmonary fibrosis. In this sense, early detection of pulmonary fibrosis is shifting toward a combination of imaging, genetic markers, and polygenic risk scores to identify high-risk individuals before symptoms appear [110]. While single genetic variants such as MUC5B have low predictive value in the general population due to the rarity of disease, they may be useful when applied to specific groups, such as those with family history or incidental interstitial lung abnormalities (ILAs) [111]. By integrating age, smoking history, and genetic data, clinicians aim to reduce significant diagnostic delays and implement earlier interventions [51].

Another emerging strategy under investigation is represented by the so-called “senotherapeutics”, which are drugs specifically designed to eliminate senescent cells and thereby inhibit their pro-inflammatory and profibrotic secretome, known as the Senescence-Associated Secretory Phenotype (SASP). In the fibrotic lung, senescent alveolar type II cells are recognized drivers of fibrosis. Two aging markers, clusterin (CLU) and lipocalin 2 (LCN2), appear to induce the fibroblast-to-myofibroblast transition [112].

## 10. Conclusions

The therapeutic landscape of pulmonary fibrosis has undergone a substantial transformation, moving from a broadly anti-inflammatory approach toward an increasingly mechanism-informed, although still evolving, model centered on the fibrotic niche. The established role of nintedanib and pirfenidone has provided the foundation for the development of a new generation of pathway-oriented therapies. Nerandomilast has expanded the therapeutic landscape by demonstrating efficacy in phase III trials and already receiving regulatory approval in some jurisdictions for IPF and PPF. Other agents, including inhaled treprostinil and admilparant, have generated encouraging clinical data but require careful interpretation according to indication, regulatory status, safety profile, and confirmatory evidence. Conversely, discontinued programs underscore the complexity of antifibrotic drug development and the need for long-term safety assessment.

Future ILD trials are progressively moving toward a more multidimensional assessment of treatment benefit. Although FVC decline remains the most widely accepted and robust marker of disease progression, the integration of patient-reported outcomes, hospitalization rates, acute exacerbations, mortality, and other clinically meaningful endpoints will be essential to determine whether emerging therapies translate into tangible benefits for patients. Overall, the integration of molecular endotyping, refinement of combination strategies, and development of mechanistically complementary agents may pave the way toward a more individualized therapeutic approach aimed at stabilizing lung function, reducing disease burden, and improving quality of life in patients with progressive fibrotic lung diseases.

## Figures and Tables

**Table 1:** Randomized clinical trials of approved antifibrotic agents in idiopathic pulmonary fibrosis and their extension trials in progressive fibrosing interstitial lung diseases.

**Idiopathic Pulmonary Fibrosis**				
**Clinical Trial**	**Drug**	**Study Design**	**Primary Endpoint**	**Results**
CAPACITY (1 and 2)	Pirfenidone	Phase 3, randomized, double-blind, and placebo-controlled (72 weeks)	Change from baseline in percent predicted FVC.	Pirfenidone significantly reduced the decline in FVC. Pooled data showed a reduction in disease progression.
ASCEND	Pirfenidone	Phase 3, randomized, double-blind, and placebo-controlled (52 weeks)	Change from baseline in percent predicted FVC.	Confirmed the findings of CAPACITY, showing a significant reduction in FVC decline. Pooled analysis with CAPACITY demonstrated a reduction in all-cause mortality.
INPULSIS (1 and 2)	Nintedanib	Phase 3, randomized, double-blind, and placebo-controlled (52 weeks)	Annual rate of decline in FVC (mL/year).	Both trials met the primary endpoint, showing that Nintedanib slowed FVC decline by ~50% compared to placebo.
FIBRONEER-IPF	Nerandomilast	Phase 3, randomized, double-blind, and placebo-controlled (52 weeks)	Absolute change from baseline in FVC (mL) at week 52.	Met the primary endpoint, showing a statistically significant reduction in FVC decline vs. placebo. Efficacy was consistent both as monotherapy and as add-on to current antifibrotics.
**Non-IPF Progressive Fibrosing Interstitial Lung Diseases**				
**Clinical Trial**	**Drug**	**Study Design**	**Primary Endpoint**	**Results**
INBUILD	Nintedanib	Phase 3, randomized, double-blind, and placebo-controlled (52 weeks)	Annual rate of decline in FVC (mL/year) over 52 weeks.	Met the primary endpoint. Nintedanib reduced the rate of FVC decline by 57% across the entire population, regardless of the fibrotic pattern (UIP-like or others).
RELIEF	Pirfenidone	Phase 2b, randomized, double-blind, and placebo-controlled (48 weeks)	Absolute change in percent predicted FVC from baseline to week 48.	Terminated early for futility. Slow recruitment (making the study underpowered to reach the primary endpoint). Failed to meet primary endpoint.
FIBRONEER-ILD	Nerandomilast	Phase 3, randomized, double-blind, and placebo-controlled (52 weeks)	Absolute change from baseline in FVC (mL) at week 52.	Met the primary endpoint. Nerandomilast significantly reduced FVC decline vs. placebo (differences of 68.8 mL and 44.9 mL for the tested doses compare to the placebo arm).

IPF, idiopathic pulmonary fibrosis; ILD, interstitial lung disease; FVC, forced vital capacity; UIP, usual interstitial pneumonia; mL, milliliters.

**Table 2:** Phase II and III clinical trials on pathway-driven emerging therapies.

Molecule	Mechanism of Action	Disease	Trial Name/Clinicaltrials.gov Identifier	Phase/Duration	Status	Number of Patients	Administration	Primary Endpoint	Adverse Events
Admilparant	LPA-r1 antagonist	IPF	NCT04308681	2/26 weeks	Completed with results	276	Oral	Change From Baseline in Percent Predicted Forced Vital Capacity (ppFVC) in IPF Participants.	Diarrhea
		PPF				123			
		IPF	ALOFT-IPF/NCT06003426	3/52 weeks	Active, not yet recruiting	1255		Number of participants that experience spontaneous syncopal events (at 4 weeks). Absolute change from baseline in forced vital capacity (FVC) measured in mL.	
		PPF	ALOFT-PPF/NCT06025578			1092			
Treprostinil	Synthetic analog of Prostacyclin	PH associated with ILD including CPFE	INCREASE/NCT02630316	2/3/16 weeks	Completed with results	326	Inhaled	Change in 6MWD Measured at Peak Exposure From Baseline to Week 16.	Cough, headache, dyspnea, etc.
			INCREASE OLE/NCT02633293	2/3/124 weeks	Terminated	243		Change in Peak 6-min Walk Distance (6MWD) From Baseline through Week 124 in RIN-PH-202.	
		IPF	TETON 2/NCT05255991	3/52 weeks	Completed with results	597		Change in Absolute FVC from Baseline to Week 52.	Cough
		IPF	TETON 1/NCT04708782		Completed with results	598			
		PPF	TETON-PPF/NCT05943535		Recruiting	698			
		IPF or PPF	TETON-OLE/NCT04905693	3/up to 6 years	Enrolling by invitation	1850		Long-term safety and tolerability of inhaled treprostinil in subjects with IPF or PPF.	
Bexotegrast	Dual integrin inhibitor (αvβ6 and αvβ1)	IPF	INTEGRIS-IPF/NCT04396756	2/up to 12 weeks	Completed with results	120	Oral	Number of Participants With Treatment-Emergent Adverse Events or Serious TEAS at 4, 12 and 48 weeks.	Diarrhea
			NCT05621252	2a/12 weeks	Completed with results	10		Change from Baseline in top quartile whole lung PET standardized uptake value (SUV) following 12 weeks of treatment with bexotegrast.	
			IPF-201/NCT04072315	2a	Completed with results	9		Number of Participants With a Predicted Effect on αVβ6 PET in Lungs After Administration of Drug.	
			BEACON-IPF/NCT06097260	2/52 weeks	Terminated early (safety concerns)	320		Change from baseline in absolute FVC (mL) at Week 52	
Sufenidone	Pyridone derivative	IPF	NCT06125327	2/3/52 weeks	Recruiting	210	Oral	Annual Rate of Decline in FVC Over 52 Weeks.	
Deupirfinidone	Deutereated formulation of pirfenidone	IPF	ELEVATE/NCT05321420	2/26 weeks	Active, not recruiting	240	Oral	Rate of decline in Forced Vital Capacity over 26 weeks	
			SURPASS-IPF/NCT07284602	3/52 weeks	Active, not recruiting	1100		Absolute change in FVC measured in mL from baseline to week 52	
Anlotinib	Tyrosine kinase inhibitor	IPF/PF-ILDs	NCT05828953	2/3/24–52 weeks	Recruiting	30	Oral	Change in FVC	

LPA-r1, lysophosphatidic acid receptor 1; IPF, idiopathic pulmonary fibrosis; PPF, progressive pulmonary fibrosis; PH, pre-capillary pulmonary hypertension; ILD, interstitial lung disease; CPFE, combined pulmonary fibrosis and emphysema; 6MWD, 6 min walking distance; TEAS, treatment-emergent adverse events; PET, positron emission tomography; SUV, standardized uptake value; FVC, forced vital capacity; ppFVC, percent predicted forced vital capacity; PF-ILD, progressive fibrosing interstitial lung disease. RIN-PH-202: INCREASE open label extension.

## Data Availability

Data sharing is not applicable.

## References

[1] Wijsenbeek M., Cottin V. (2020). Spectrum of Fibrotic Lung Diseases. N. Engl. J. Med..

[2] Wijsenbeek M., Suzuki A., Maher T.M. (2022). Interstitial Lung Diseases. Lancet.

[3] Maher T.M. (2024). Interstitial Lung Disease: A Review. JAMA.

[4] Johannson K.A., Chaudhuri N., Adegunsoye A., Wolters P.J. (2021). Treatment of Fibrotic Interstitial Lung Disease: Current Approaches and Future Directions. Lancet.

[5] Moss B.J., Ryter S.W., Rosas I.O. (2022). Pathogenic Mechanisms Underlying Idiopathic Pulinary Fibrosis. Annu. Rev. Pathol..

[6] Fernandez I.E., Eickelberg O. (2012). New Cellular and Molecular Mechanisms of Lung Injury and Fibrosis in Idiopathic Pulmonary Fibrosis. Lancet.

[7] Lederer D.J., Martinez F.J. (2018). Idiopathic Pulmonary Fibrosis. N. Engl. J. Med..

[8] Richeldi L., Collard H.R., Jones M.G. (2017). Idiopathic Pulmonary Fibrosis. Lancet.

[9] Raghu G., Remy-Jardin M., Richeldi L. (2022). Idiopathic Pulmonary Fibrosis (An Update) and Progressive Pulmonary Fibrosis in Adults: An Official ATS/ERS/JRS/ALAT Clinical Practice Guideline. Am. J. Respir. Crit. Care Med..

[10] Wang J., Chao J. (2025). Epithelial Cell Dysfunction in Pulmonary Fibrosis: Mechanisms, Interactions, and Emerging Therapeutic Targets. Pharmaceuticals.

[11] Mutsaers S.E., Miles T., Prele C.M., Hoyne G.F. (2023). Emerging Role of Immune Cells as Drivers of Pulmonary Fibrosis. Pharmacol. Ther..

[12] Hoffman E.T., Shah A., Barboza W.R. (2025). Aberrant Intermediate Alveolar Epithelial Cells Promote Pathogenic Activation of Lung Fibroblasts in Preclinical Fibrosis Models. Nat. Commun..

[13] Morse C., Tabib T., Sembrat J. (2019). Proliferating SPP1/MERTK-Expressing Macrophages in Idiopathic Pulmonary Fibrosis. Eur. Respir. J..

[14] Kishore A., Petrek M. (2021). Roles of Macrophage Polarization and Macrophage-Derived MiRNAs in Pulmonary Fibrosis. Front. Immunol..

[15] Ge Z., Chen Y., Ma L., Hu F., Xie L. (2024). Macrophage Polarization and Its Impact on Idiopathic Pulmonary Fibrosis. Front. Immunol..

[16] Isshiki T., Vierhout M., Naiel S. (2023). Therapeutic Strategies Targeting Pro-Fibrotic Macrophages in Interstitial Lung Disease. Biochem. Pharmacol..

[17] Yang X., Liu Z., Zhou J. (2024). SPP1 Promotes the Polarization of M2 Macrophages Through the Jak2/Stat3 Signaling Pathway and Accelerates the Progression of Idiopathic Pulmonary Fibrosis. Int. J. Mol. Med..

[18] Hou J., Ji J., Chen X. (2021). Alveolar Epithelial Cell-Derived Sonic Hedgehog Promotes Pulmonary Fibrosis Through OPN-Dependent Alternative Macrophage Activation. FEBS J..

[19] Ji J., Zheng S., Liu Y. (2023). Increased Expression of OPN Contributes to Idiopathic Pulmonary Fibrosis and Indicates a Poor Prognosis. J. Transl. Med..

[20] Ligresti G., Raslan A.A., Hong J. (2023). Mesenchymal Cells in the Lung: Evolving Concepts and Their Role in Fibrosis. Gene.

[21] Yao L., Zhou Y., Li J. (2021). Bidirectional Epithelial-Mesenchymal Crosstalk Provides Self-Sustaining Profibrotic Signals in Pulmonary Fibrosis. J. Biol. Chem..

[22] Younesi F.S., Miller A.E., Barker T.H., Rossi F.M.V., Hinz B. (2024). Fibroblast and Myofibroblast Activation in Normal Tissue Repair and Fibrosis. Nat. Rev. Mol. Cell Biol..

[23] Yamaguchi M., Hirai S., Tanaka Y. (2017). Fibroblastic Foci, Covered with Alveolar Epithelia Exhibiting Epithelial-Mesenchymal Transition, Destroy Alveolar Septa by Disrupting Blood Flow in Idiopathic Pulmonary Fibrosis. Lab. Investig..

[24] Singh P., Edjah S., Shi W., Madala S. (2025). Emerging Concepts in Fibroblast Biology and Progressive Pulmonary Fibrosis. Semin. Respir. Crit. Care Med..

[25] Herrera J.A., Dingle L., Montero M.A. (2022). The UIP/IPF Fibroblastic Focus Is a Collagen Biosynthesis Factory Embedded in a Distinct Extracellular Matrix. JCI Insight.

[26] Guillotin D., Taylor A.R., Plate M. (2021). Transcriptome Analysis of IPF Fibroblastic Foci Identifies Key Pathways Involved in Fibrogenesis. Thorax.

[27] Gupta D., Kumar A., Mandloi A., Shenoy V. (2021). Renin Angiotensin Aldosterone System in Pulmonary Fibrosis: Pathogenesis to Therapeutic Possibilities. Pharmacol. Res..

[28] Yao C., Guan X., Carraro G. (2021). Senescence of Alveolar Type 2 Cells Drives Progressive Pulmonary Fibrosis. Am. J. Respir. Crit. Care Med..

[29] Enomoto Y., Katsura H., Fujimura T. (2023). Autocrine TGF-β-Positive Feedback in Profibrotic AT2-Lineage Cells Plays a Crucial Role in Non-Inflammatory Lung Fibrogenesis. Nat. Commun..

[30] Craig V.J., Zhang L., Hagood J.S., Owen C.A. (2015). Matrix Metalloproteinases as Therapeutic Targets for Idiopathic Pulmonary Fibrosis. Am. J. Respir. Cell Mol. Biol..

[31] Frangogiannis N. (2020). Transforming Growth Factor-βin Tissue Fibrosis. J. Exp. Med..

[32] Noskovicova N., Petrek M., Eickelberg O., Heinzelmann K. (2015). Platelet-Derived Growth Factor Signaling in the Lung: From Lung Development and Disease to Clinical Studies. Am. J. Respir. Cell Mol. Biol..

[33] Wermuth P.J., Li Z., Mendoza F.A., Jimenez S.A. (2016). Stimulation of Transforming Growth Factor-β1-Induced Endothelial-to-Mesenchymal Transition and Tissue Fibrosis by Endothelin-1. PLoS ONE.

[34] Ross B., D’Orleans-Juste P., Giaid A. (2010). Potential Role of Endothelin-1 in Pulmonary Fibrosis: From the Bench to the Clinic. Am. J. Respir. Cell Mol. Biol..

[35] Chen F., Lyu L., Xing C. (2025). The Pivotal Role of TGF-β/Smad Pathway in Fibrosis Pathogenesis and Treatment. Front. Oncol..

[36] Ong C.H., Tham C.L., Harith H.H., Firdaus N., Israf D.A. (2021). TGF-β-Induced Fibrosis: A Review on the Underlying Mechanism and Potential Therapeutic Strategies. Eur. J. Pharmacol..

[37] Antoniades H.N., Bravo M.A., Avila R.E. (1990). Platelet-Derived Growth Factor in Idiopathic Pulmonary Fibrosis. J. Clin. Investig..

[38] Bozyk P.D., Moore B.B. (2011). Prostaglandin E2 and the Pathogenesis of Pulmonary Fibrosis. Am. J. Respir. Cell Mol. Biol..

[39] Mukherjee S., Sheng W., Michkov A. (2019). Prostaglandin E Inhibits Profibrotic Function of Human Pulmonary Fibroblasts by Disrupting Ca Signaling. Am. J. Physiol. Lung Cell. Mol. Physiol..

[40] Isshiki T., Naiel S., Vierhout M. (2024). Therapeutic Strategies to Target Connective Tissue Growth Factor in Fibrotic Lung Diseases. Pharmacol. Ther..

[41] Yanagihara T., Tsubouchi K., Gholiof M. (2022). Connective-Tissue Growth Factor Contributes to TGF-β1-Induced Lung Fibrosis. Am. J. Respir. Cell Mol. Biol..

[42] Yang J., Velikoff M., Canalis E., Horowitz J.C., Kim K.K. (2014). Activated Alveolar Epithelial Cells Initiate Fibrosis Through Autocrine and Paracrine Secretion of Connective Tissue Growth Factor. Am. J. Physiol. Lung Cell. Mol. Physiol..

[43] Barratt S.L., Blythe T., Jarrett C. (2017). Differential Expression of VEGF-Axxx Isoforms Is Critical for Development of Pulmonary Fibrosis. Am. J. Respir. Crit. Care Med..

[44] Farkas L., Farkas D., Ask K. (2009). VEGF Ameliorates Pulmonary Hypertension Through Inhibition of Endothelial Apoptosis in Experimental Lung Fibrosis in Rats. J. Clin. Investig..

[45] Andrianifahanana M., Wilkes M.C., Gupta S.K. (2013). Profibrotic TGF-β Responses Require the Cooperative Action of PDGF and ErbB Receptor Tyrosine Kinases. FASEB J..

[46] Gu H., Mickler E.A., Cummings O.W. (2014). Crosstalk Between TGF-β1 and Complement Activation Augments Epithelial Injury in Pulmonary Fibrosis. FASEB J..

[47] Oikonomou N., Mouratis M.-A., Tzouvelekis A. (2012). Pulmonary Autotaxin Expression Contributes to the Pathogenesis of Pulmonary Fibrosis. Am. J. Respir. Cell Mol. Biol..

[48] Tager A.M., LaCamera P., Shea B.S. (2008). The Lysophosphatidic Acid Receptor LPA1 Links Pulmonary Fibrosis to Lung Injury by Mediating Fibroblast Recruitment and Vascular Leak. Nat. Med..

[49] Neighbors M., Li Q., Zhu S.J. (2023). Bioactive Lipid Lysophosphatidic Acid Species Are Associated with Disease Progression in Idiopathic Pulmonary Fibrosis. J. Lipid Res..

[50] Alder J.K., Armanios M. (2022). Telomere-Mediated Lung Disease. Physiol. Rev..

[51] Adegunsoye A., Kropski J.A., Behr J. (2024). Genetics and Genomics of Pulmonary Fibrosis: Charting the Molecular Landscape and Shaping Precision Medicine. Am. J. Respir. Crit. Care Med..

[52] Newton C.A., Oldham J.M., Applegate C. (2022). The Role of Genetic Testing in Pulmonary Fibrosis: A Perspective from the Pulmonary Fibrosis Foundation Genetic Testing Work Group. Chest.

[53] Moore C., Blumhagen R.Z., Yang I. (2019). V Resequencing Study Confirms That Host Defense and Cell Senescence Gene Variants Contribute to the Risk of Idiopathic Pulmonary Fibrosis. Am. J. Respir. Crit. Care Med..

[54] Juge P.A., Lee J.S., Ebstein E. (2018). MUC5B Promoter Variant and Rheumatoid Arthritis with Interstitial Lung Disease. N. Engl. J. Med..

[55] Barnes P.J., Baker J., Donnelly L.E. (2019). Cellular Senescence as a Mechanism and Target in Chronic Lung Diseases. Am. J. Respir. Crit. Care Med..

[56] Katzen J., Beers M.F. (2020). Contributions of Alveolar Epithelial Cell Quality Control to Pulmonary Fibrosis. J. Clin. Investig..

[57] Lee J.S., La J., Aziz S. (2021). Molecular Markers of Telomere Dysfunction and Senescence Are Common Findings in the Usual Interstitial Pneumonia Pattern of Lung Fibrosis. Histopathology.

[58] Wang X.C., Song K., Tu B. (2023). New Aspects of the Epigenetic Regulation of EMT Related to Pulmonary Fibrosis. Eur. J. Pharmacol..

[59] Raghu G., Anstrom K.J., King T.E., Lasky J.A., Martinez F.J., Idiopathic Pulmonary Fibrosis Clinical Research Network (2012). Prednisone, Azathioprine, and N-Acetylcysteine for Pulmonary Fibrosis. N. Engl. J. Med..

[60] Noble P.W., Albera C., Bradford W.Z., Costabel U., Glassberg M.K., Kardatzke D., King T.E., Lancaster L., Sahn S.A., Szwarcberg J. (2011). Pirfenidone in Patients with Idiopathic Pulmonary Fibrosis (CAPACITY): Two Randomised Trials. Lancet.

[61] King T.E., Bradford W.Z., Castro-Bernardini S., Fagan E.A., Glaspole I., Glassberg M.K., Gorina E., Hopkins P.M., Kardatzke D., Lancaster L. (2014). A Phase 3 Trial of Pirfenidone in Patients with Idiopathic Pulmonary Fibrosis. N. Engl. J. Med..

[62] Richeldi L., du Bois R.M., Raghu G., Azuma A., Brown K.K., Costabel U., Cottin V., Flaherty K.R., Hansell D.M., Inoue Y. (2014). Efficacy and Safety of Nintedanib in Idiopathic Pulmonary Fibrosis. N. Engl. J. Med..

[63] Chianese M., Screm G., Salton F. (2024). Pirfenidone and Nintedanib in Pulmonary Fibrosis: Lights and Shadows. Pharmaceuticals.

[64] Distler O., Highland K.B., Gahlemann M. (2019). Nintedanib for Systemic Sclerosis-Associated Interstitial Lung Disease. N. Engl. J. Med..

[65] Highland K.B., Distler O., Kuwana M. (2021). Efficacy and Safety of Nintedanib in Patients with Systemic Sclerosis-Associated Interstitial Lung Disease Treated with Mycophenolate: A Subgroup Analysis of the SENSCIS Trial. Lancet Respir. Med..

[66] Allanore Y., Vonk M.C., Distler O. (2022). Continued Treatment with Nintedanib in Patients with Systemic Sclerosis-Associated Interstitial Lung Disease: Data from SENSCIS-ON. Ann. Rheum. Dis..

[67] Flaherty K.R., Wells A.U., Cottin V., Devaraj A., Walsh S.L.F., Inoue Y., Richeldi L., Kolb M., Tetzlaff K., Stowasser S. (2019). Nintedanib in Progressive Fibrosing Interstitial Lung Diseases. N. Engl. J. Med..

[68] Dixon G., Hague S., Mulholland S. (2024). Real-World Experience of Nintedanib for Progressive Fibrosing Interstitial Lung Disease in the UK. ERJ Open Res..

[69] Mondoni M., Varone F., Luppi F. (2025). Effectiveness of Nintedanib in Progressive Pulmonary Fibrosis Assessed by Progression Criteria: An Italian, Observational, Multicenter Study. Lung.

[70] Muscato G., Libra A., Reina C. (2026). Nintedanib for Progressive Pulmonary Fibrosis in Real-World Setting: An Observational Study Comparing Outcomes with an IPF Cohort. BMC Pulm. Med..

[71] Behr J., Prasse A., Kreuter M. (2021). Pirfenidone in Patients with Progressive Fibrotic Interstitial Lung Diseases Other Than Idiopathic Pulmonary Fibrosis (RELIEF): A Double-Blind, Randomised, Placebo-Controlled, Phase 2b Trial. Lancet Respir. Med..

[72] Solomon J.J., Danoff S.K., Woodhead F.A. (2023). Safety, Tolerability, and Efficacy of Pirfenidone in Patients with Rheumatoid Arthritis-Associated Interstitial Lung Disease: A Randomized, Double-Blind, Placebo-Controlled, Phase 2 Study. Lancet Respir. Med..

[73] Juge P.A., Hayashi K., McDermott G.C. (2024). Effectiveness and Tolerability of Antifibrotics in Rheumatoid Arthritis-Associated Interstitial Lung Disease. Semin. Arthritis Rheum..

[74] Liang M., Matteson E.L., Abril A., Distler J.H.W. (2022). The Role of Antifibrotics in the Treatment of Rheumatoid Arthritis-Associated Interstitial Lung Disease. Ther. Adv. Musculoskelet. Dis..

[75] Johnson S.R., Bernstein E.J., Bolster M.B. (2024). 2023 American College of Rheumatology (ACR)/American College of Chest Physicians (CHEST) Guideline for the Treatment of Interstitial Lung Disease in People with Systemic Autoimmune Rheumatic Diseases. Arthritis Care Res..

[76] Raghu G., Montesi S.B., Silver R.M. (2024). Treatment of Systemic Sclerosis-Associated Interstitial Lung Disease: Evidence-Based Recommendations. An Official American Thoracic Society Clinical Practice Guideline. Am. J. Respir. Crit. Care Med..

[77] Antoniou K.M., Distler O., Gheorghiu A.M. (2026). ERS/EULAR Clinical Practice Guidelines for Connective Tissue Disease-Associated Interstitial Lung Disease. Eur. Respir. J..

[78] Spagnolo P., Maher T.M. (2024). The Future of Clinical Trials in Idiopathic Pulmonary Fibrosis. Curr. Opin. Pulm. Med..

[79] Maher T.M., Ford P., Brown K.K. (2023). Ziritaxestat, a Novel Autotaxin Inhibitor, and Lung Function in Idiopathic Pulmonary Fibrosis: The ISABELA 1 and 2 Randomized Clinical Trials. JAMA.

[80] Richeldi L., Schiffman C., Behr J. (2024). Zinpentraxin Alfa for Idiopathic Pulmonary Fibrosis: The Randomized Phase III STARSCAPE Trial. Am. J. Respir. Crit. Care Med..

[81] Raghu G., Richeldi L., Fernandez Perez E.R. (2024). Pamrevlumab for Idiopathic Pulmonary Fibrosis: The ZEPHYRUS-1 Randomized Clinical Trial. JAMA.

[82] Richeldi L., Azuma A., Cottin V. (2025). Nerandomilast in Patients with Idiopathic Pulmonary Fibrosis. N. Engl. J. Med..

[83] Maher T.M., Assassi S., Azuma A. (2025). Nerandomilast in Patients with Progressive Pulmonary Fibrosis. N. Engl. J. Med..

[84] Richeldi L., Azuma A., Cottin V. (2022). Trial of a Preferential Phosphodiesterase 4B Inhibitor for Idiopathic Pulmonary Fibrosis. N. Engl. J. Med..

[85] Kolb M., Crestani B., Maher T.M. (2023). Phosphodiesterase 4B Inhibition: A Potential Novel Strategy for Treating Pulmonary Fibrosis. Eur. Respir. Rev..

[86] Oldham J.M., Azuma A., Kreuter M. (2026). Nerandomilast in Idiopathic Pulmonary Fibrosis: Data from the Whole Follow-Up Period of the FIBRONEER-IPF Trial. Am. J. Respir. Crit. Care Med..

[87] Reininger D., Wolf F., Mayr C.H. (2025). Insights into the Cellular and Molecular Mechanisms Behind the Antifibrotic Effects of Nerandomilast. Am. J. Respir. Cell Mol. Biol..

[88] Peters-Golden M., Fortier S.M. (2026). Mechanistic Basis for the Antifibrotic Actions of CAMP-Based Therapies. Eur. Respir. Rev..

[89] Mondoni M., Rinaldo R., Ryerson C.J. (2024). Vascular Involvement in Idiopathic Pulmonary Fibrosis. ERJ Open Res..

[90] Tirelli C., Pesenti C., Miozzo M., Mondoni M., Fontana L., Centanni S. (2022). The Genetic and Epigenetic Footprint in Idiopathic Pulmonary Fibrosis and Familial Pulmonary Fibrosis: A State-of-the-Art Review. Diagnostics.

[91] Denis A., Tsiri P., Guiot J., Tzouvelekis A. (2025). A New Era in the Treatment of Progressive Fibrosing Interstitial Lung Diseases. Breathe.

[92] Corte T.J., Behr J., Cottin V. (2025). Efficacy and Safety of Admilparant, an LPA1 Antagonist, in Pulmonary Fibrosis: A Phase 2 Randomized Clinical Trial. Am. J. Respir. Crit. Care Med..

[93] Kreuter M., Maher T.M., Wuyts W.A. (2025). Effect of Admilparant, a Lysophosphatidic Acid Receptor 1 Antagonist, on Disease Progression in Pulmonary Fibrosis. Chest.

[94] Lindegaard Pedersen M., Kruger M., Grimm D., Infanger M., Wehland M. (2020). The Prostacyclin Analogue Treprostinil in the Treatment of Pulmonary Arterial Hypertension. Basic Clin. Pharmacol. Toxicol..

[95] Waxman A., Restrepo-Jaramillo R., Thenappan T. (2021). Inhaled Treprostinil in Pulmonary Hypertension Due to Interstitial Lung Disease. N. Engl. J. Med..

[96] Nathan S.D., Waxman A., Rajagopal S. (2021). Inhaled Treprostinil and Forced Vital Capacity in Patients with Interstitial Lung Disease and Associated Pulmonary Hypertension: A Post-Hoc Analysis of the INCREASE Study. Lancet Respir. Med..

[97] Nathan S.D., Johri S., Joly J.M. (2024). Survival Analysis from the INCREASE Study in PH-ILD: Evaluating the Impact of Treatment Crossover on Overall Mortality. Thorax.

[98] Waxman A., Restrepo-Jaramillo R., Thenappan T. (2023). Long-Term Inhaled Treprostinil for Pulmonary Hypertension Due to Interstitial Lung Disease: INCREASE Open-Label Extension Study. Eur. Respir. J..

[99] Nathan S.D., Smith P., Deng C., De Salvo M., Wuyts W., Pavie-Gallegos J., Song J.W., Kramer M.R., King C.S., Mackintosh J.A. (2026). Inhaled Treprostinil for Idiopathic Pulmonary Fibrosis. N. Engl. J. Med..

[100] Decaris M.L., Schaub J.R., Chen C. (2021). Dual Inhibition of αvβ6 and αvβ1 Reduces Fibrogenesis in Lung Tissue Explants from Patients with IPF. Respir. Res..

[101] Lancaster L., Cottin V., Ramaswamy M. (2024). Bexotegrast in Patients with Idiopathic Pulmonary Fibrosis: The INTEGRIS-IPF Clinical Trial. Am. J. Respir. Crit. Care Med..

[102] Montesi S.B., Cosgrove G.P., Turner S.M. (2025). Dual αvβ6 and αvβ1 Inhibition over 12 Weeks Reduces Active Type I Collagen Deposition in Individuals with Idiopathic Pulmonary Fibrosis: A Phase 2, Double-Blind, Placebo-Controlled Clinical Trial. Am. J. Respir. Crit. Care Med..

[103] Mooney J.J., Jacobs S., Lefebvre E.A. (2025). Bexotegrast Shows Dose-Dependent Integrin αvβ6 Receptor Occupancy in Lungs of Participants with Idiopathic Pulmonary Fibrosis: A Phase 2, Open-Label Clinical Trial. Ann. Am. Thorac. Soc..

[104] Wuyts W.A., Lancaster L., Maher T.M. (2026). Bexotegrast for Treatment of Idiopathic Pulmonary Fibrosis (BEACON-IPF): Study Protocol for a Multinational, Phase 2b/3, Double-Blind, Randomised, Multicentre, Controlled Trial. BMJ Open Respir. Res..

[105] Liu Y., Chen X., Tang H. (2025). Safety, Tolerability, and Pharmacokinetics of SC1011 (Sufenidone), a Novel Antifibrotic Small Molecule, in Phase 1 Studies in Healthy Subjects. Clin. Transl. Sci..

[106] Chen M.C., Korth C.C., Harnett M.D., Elenko E., Lickliter J.D. (2022). A Randomized Phase 1 Evaluation of Deupirfenidone, a Novel Deuterium-Containing Drug Candidate for Interstitial Lung Disease and Other Inflammatory and Fibrotic Diseases. Clin. Pharmacol. Drug Dev..

[107] Maher T.M., Bergna M.A., Hajari Case A. (2025). Deupirfenidone Compared to Placebo and Pirfenidone in Idiopathic Pulmonary Fibrosis: ELEVATE IPF Phase 2b Trial. Am. J. Respir. Crit. Care Med..

[108] Alonso-Gonzalez A., Tosco-Herrera E., Molina-Molina M., Flores C. (2023). Idiopathic Pulmonary Fibrosis and the Role of Genetics in the Era of Precision Medicine. Front. Med..

[109] Oldham J.M., Noth I., Martinez F.J. (2016). Pharmacogenetics and Interstitial Lung Disease. Curr. Opin. Pulm. Med..

[110] Raghu G. (2017). Pharmacotherapy for Idiopathic Pulmonary Fibrosis: Current Landscape and Future Potential. Eur. Respir. Rev..

[111] Moll M., Peljto A.L., Kim J.S. (2023). A Polygenic Risk Score for Idiopathic Pulmonary Fibrosis and Interstitial Lung Abnormalities. Am. J. Respir. Crit. Care Med..

[112] Zhang W., Xia T., Zhang Q. (2026). The Role of Age-Related Genes in Idiopathic Pulmonary Fibrosis and Molecular Docking Analysis of Their Drug Targets. Front. Immunol..

